# Conspicuous Female Ornamentation and Tests of Male Mate Preference in Threespine Sticklebacks (*Gasterosteus aculeatus*)

**DOI:** 10.1371/journal.pone.0120723

**Published:** 2015-03-25

**Authors:** Daniel Shane Wright, Michele E. R. Pierotti, Howard D. Rundle, Jeffrey S. McKinnon

**Affiliations:** 1 Department of Biology and Center for Biodiversity, East Carolina University, Greenville, North Carolina, United States of America; 2 Department of Biology, University of Ottawa, Ottawa, Ontario, Canada; Utrecht University, NETHERLANDS

## Abstract

Sexual selection drives the evolution of exaggerated male ornaments in many animal species. Female ornamentation is now acknowledged also to be common but is generally less well understood. One example is the recently documented red female throat coloration in some threespine stickleback (*Gasterosteus aculeatus*) populations. Although female sticklebacks often exhibit a preference for red male throat coloration, the possibility of sexual selection on female coloration has been little studied. Using sequential and simultaneous mate choice trials, we examined male mate preferences for female throat color, as well as pelvic spine color and standard length, using wild-captured threespine sticklebacks from the Little Campbell River, British Columbia. In a multivariate analysis, we found no evidence for a population-level mate preference in males, suggesting the absence of directional sexual selection on these traits arising from male mate choice. Significant variation was detected among males in their preference functions, but this appeared to arise from differences in their mean responsiveness across mating trials and not from variation in the strength (i.e., slope) of their preference, suggesting the absence of individual-level preferences as well. When presented with conspecific intruder males, male response decreased as intruder red throat coloration increased, suggesting that males can discriminate color and other aspects of phenotype in our experiment and that males may use these traits in intrasexual interactions. The results presented here are the first to explicitly address male preference for female throat color in threespine sticklebacks.

## Introduction

There is now abundant evidence that sexual selection underlies the evolution of exaggerated male ornamentation (traits showing little to no function outside of social interactions) in many animal species[1]. Perhaps surprisingly, female ornamentation also varies greatly across taxa, and is sometimes as conspicuous as in males. Yet the presence of similar ornamental traits in the females of many animal species remains a poorly understood phenomenon. It is widely thought that male reproductive success is limited principally by the number of mates that can be obtained [2], making male ornamentation important for attracting females and/or intimidating rival males. Females on the other hand, should rarely need to compete for mates and thus sexual selection favoring female ornamentation should be weaker or absent. However, females in a wide variety of species are now known to possess such adornments[3], including birds[4–6], reptiles [7,8], insects[9], and fish[10–12].

One hypothesis to explain female ornaments is that they result from intersexual genetic correlation with sexually selected male traits[13]. It is only by selection, usually natural selection, acting against the female trait that such a correlation is broken and females lose ornaments favored in males, and this loss may be slow or incomplete[6]. A second, competing hypothesis involves the notion of selection acting directly on females, for example by way of male mating preferences, to favor ornamentation[14]. In a noteworthy case study, Amundsen and Forsgren[15] found that male two-spotted gobies prefer females with more intense yellow-orange belly coloration. Related work in birds has shown similar (although mixed) results with males often preferring more ornamented females[16]. Male preferences can be attributed in part to differential parental investment[17]; mating systems involving paternal care can facilitate the evolution of female ornaments (i.e. male preference for female traits that serve as an indicator of mate quality). As noted by Edward and Chapman[18], male mate choice (and ensuing female ornamentation) seems to be a more common phenomenon than previously suggested.

For nearly one hundred years, the threespine stickleback (*Gasterosteus aculeatus*) has been studied as a model system of behavior and evolution, most recently with particular emphasis on speciation and sexual selection[19–21]. This species, found throughout much of the Northern Hemisphere, resides in both marine and freshwater environments and takes on an array of sizes and forms. Typically, males develop red coloration of the throat and anterior lateral areas, as well as bright blue eyes, during the breeding season between March and July[22]. The male will establish and defend a nest, courting nearby females. Receptive females respond by approaching the male with a ‘head-up’ display (which also exposes any throat coloration). After mating, a male remains with the nest, potentially mating with additional females and defending the nest until the eggs have hatched (as well as defending the fry initially: [22]). Ter Pelkwijk and Tinbergen[23] found that female sticklebacks show a preference for male red coloration, noting that females continually followed red-colored dummy males to nesting sites. Subsequent work has confirmed female preference for male nuptial color in several populations as well as the utility of throat coloration in identifying higher quality mates[24,25].

**Fig 1:**
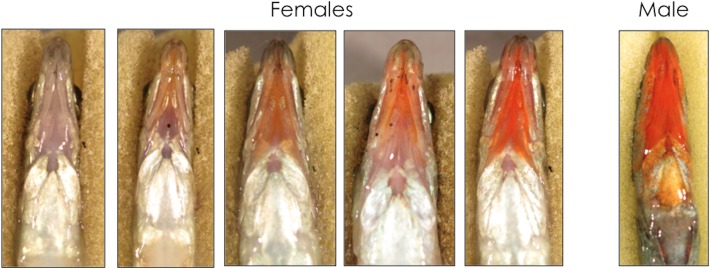
Variation in female throat color. Variation in stream resident female throat coloration alongside a typical male.

McKinnon *et al*. [26] were the first to document that in the Little Campbell River in Southwestern British Columbia, female threespine sticklebacks commonly possess the conspicuous male trait, red throat coloration (Fig. 1). These (upper) stream-resident females not only closely resemble their male counterparts, but also differ dramatically from nearby populations. Lower stream (anadromous) females show almost no throat coloration and marine/anadromous populations are generally held to represent the ancestral state in this species[27]. Males from the two populations display no consistent difference in the intensity of their throat ornamentation[26] and in fact male coloration is more extensive in the anadromous population[26]. Von Hippel[28] documented a similar pattern in a California population in which some females displayed conspicuous red throat coloration. In an Eastern United States population, gravid female sticklebacks develop a distinct pattern, lateral vertical barring, and males court females with this pattern preferentially[29]. In a Norwegian stickleback population, both males and females have been shown to possess red pelvic spine coloration (also see[12]) and males direct more courtship behavior toward females with drab spine color[30], who in turn possess eggs with higher carotenoid levels[31]. Recent work has also shown that UV patterns are important in stickleback mate choice[32,33].

The presence of female throat color, potentially driven by male preferences for female traits, is not unexpected in sticklebacks in light of substantial male parental investment[27]. Repeated bouts of care and starvation throughout the breeding season severely limit a male’s reproductive capacity. Late in the breeding season, males may become a limited resource, thus shifting the operational sex ratio (OSR) toward a female bias. Indeed, lake and stream observations by Kynard[34] and Mori[35], respectively, have documented courting females sometimes outnumbering courting males in the late breeding season, even to the point of several females attempting to court a single male. As noted by Kokko *et*. *al*. [36], OSR may involve both of these factors: the ratio of males to females in the mating pool and the ‘time-out’ of a given sex during each reproductive bout. Additionally, the upper and lower regions of the Little Campbell vary drastically in some environmental factors (estuarine vs. freshwater). Habitat variation, combined with a shifting OSR, could set the stage for sexual selection by males and aid in the persistence of female throat color ornamentation.

This study aims to investigate possible sexual selection by males for female threespine stickleback ornamentation. It (1) highlights the possible role of sexual selection on female ornamentation as well as allowing for (2) analysis of individual male preferences and variation among males. Using multiple preference tests, we address the question: Do males show a preference for red throat coloration in females? It is hypothesized that males will demonstrate a preference for females displaying red throat coloration, based on the knowledge that females show a preference for the same trait in males and males invest heavily in each reproductive bout. Male preference is analyzed as time spent in proximity to, and courtship behaviors directed toward, females for which throat color (as well as pelvic spine color and standard length) has been physically assessed and evaluated. Individual variation in male preference and response is examined, as it could be important in the maintenance of female polymorphisms but has been little studied[37].

## Methods

### Fish collections and nesting stimulation

Using minnow traps, fish were collected from field sites in British Columbia, Canada (Little Campbell River: BC, Canada, 49.0321N—122.657W) at the start and at the peak of breeding season (late March then late May 2010 and April then June 2011) and transported to a climate-controlled facility at East Carolina University, North Carolina. Fish were collected under the authority of the British Columbia Ministry of Environment/Natural Resource Operations (permit numbers: SU10–60709 & SU11–68627 respectively) and with permission from the Greater Vancouver Regional District Parks Department (2011). Only males collected at the start of the breeding season were used in experimental trials (to limit previous breeding experience and ensure male condition and willingness to nest). All fish were housed under approximately natural lighting, (wide-spectrum day/night cycle lighting, seasonally increasing day lengths) and temperatures (14–16°C). This study was performed with approval of the Animal Care and Use Committee of East Carolina University (Animal Use Protocol—AUP #D224).

Males demonstrating breeding coloration of blue eyes and red throat were isolated in 102L aquaria. Each tank was covered with heavy brown paper on three sides to visually isolate males from one another. For nesting material, a plastic dish containing sand and autoclaved plant material (moss) was placed at the center of the tank. Each tank had an air stone and filter running continuously to ensure proper water quality. In order to provide relatively natural lighting, a *Solux* Daylight MR16 halogen bulb was mounted at the end of the tank, opposite from where experimental females were to be presented. A Commercial Electric 5.5” clamp lamp with compact fluorescent, 60-watt black light bulb was used to supplement UV. Following a period of 24–48 hours to acclimate to his new surroundings, each male was stimulated for nesting and courtship behavior with a mixture of gravid females from a separate, but nearby stream resident population (Nicomekl River) containing females with similar throat coloration (DSW, *pers*. *obs*.). No females used in nest stimulations were used in the subsequent experimental trials. Three to four gravid females varying in throat color and body size were selected haphazardly and presented in a separate (smaller) tank located immediately adjacent to the male’s tank for 10–15 minutes each day for up to five days. To allow for olfactory cues, approximately 20mL of water was transferred from the female stimulation tank to the male test tank at the beginning of nest stimulation. If the male failed to initiate nest building after five days, gravid females (varying in throat color and body size) were introduced into the tank (free-swimming) for ten minutes/day, for up to five days. If the male still failed to begin to build his nest after ten days, he was removed, the tank reset, and a new male introduced.

### Experimental Trials

#### Sequential choice preference tests

Once a male had completed his nest and begun to display courtship behavior (zigzagging), he was isolated from female stimulation for a period of 24 hours. Over the next five days, males were sequentially presented with five same population gravid females, one per day (as discussed in[38]). Gravidity of females was determined by the presence of a swollen abdomen prior to feeding. All presentations for an individual male occurred at the same time each day, during the morning (8:00–10:00) or afternoon (16:00–18:00). Each female was used only once per male, with most females used just once and no single female more than two total times (Year 1: 155 different females used. Year 2: 140 different females used, 20 females used a second time but in unique combinations and confined to four males). Females were presented in water-sealed, UV transparent, Plexiglas containers, 7cm long and 2cm wide, approximately 30cm from the nest and oriented toward it. The dimensions of the containers allowed for the upper portion to remain out of the water and allow adequate room for the female to obtain oxygen supplied by a lightly running air stone. The base of the container was inclined at approximately 25° from the horizontal to replicate the ‘head-up’ display of female courtship, exposing throat color to isolated males. Females were lightly anesthetized with MS-222 (37.5–50mg/L) to minimize movement and its possible effect on male behavior (MS-222 treatment had no visible effect on female throat color). A single, vertical line on the back of the tank allowed for constant ‘threshold’ of proximity/interaction designation—once a male crossed this line with >50% of body length, he was considered to be in proximity to the test female, remaining in proximity until crossing the line again in the opposite direction. This line was drawn so that there was approximately equal distance (∼18cm) in front of and behind the female presentation container (because of tank construction, the female container, when placed opposite of the male’s nest, could be no closer to the tank wall than ∼18cm. An equivalent distance was marked in front of the container to ensure an equal area of interaction designation for each trial). Each presentation trial lasted 10 min and was video recorded using a *Sony Handycam* HDR-XR500V from the side of the tank. At the conclusion of each trial, females were scored for throat color and other traits (including body size and spine color, described below). Males were scored in the same manner at the conclusion of each presentation series. A total of 63 males were tested over the course of two years. Year one trials took place between May 10 and August 15, 2010 with 31 males. Year two trials took place between May 4 and August 2, 2011 with 32 males.

#### Simultaneous choice preference tests

In the second (2011) year, 24 h after the completion of the fifth sequential choice presentation, males were presented with two size-matched, gravid females differing in throat color. Females were selected so as to provide the greatest possible difference in throat coloration but still be equivalent in size. For each male, no female presented during the sequential choice trials was reused in a simultaneous choice presentation. Females were presented in the same manner as the sequential choice trials, side by side, approximately 10cm from one another. Side designation for females (left or right) was randomly determined for each trial. Simultaneous choice tests were filmed from behind the male’s nest, directly facing the two test females. All (n = 32 males) simultaneous choice trials took place during year two between May 9 and August 3, 2011.

#### Conspecific male tests

Presentation of a conspecific male serves as a ‘control’ to determine whether males: (1) discriminate characteristics of fish within the presentation container; (2) modify their behavior in response to stimuli of different sex. Males tested during the second year received a conspecific male presentation 24 h after the simultaneous choice test. Following the same protocol as the female sequential choice trials, males were presented with a same population male displaying breeding coloration.

### Trait Assessment

#### Throat color

Reflectance measurements of each fish’s throat were taken using an *Ocean Optics* Maya 2000 spectrometer and SpectraSuite software following the procedure detailed in Yong *et al*. [12]. As in Yong *et al*. [12], all reflectance measurements were standardized with a white standard, Spectralon and were taken from 2–3 throat spots (approximately 0.8 mm in diameter) deliberately chosen to yield maximum red throat chroma. To account for how the male’s eye receptors adapt independently to background light present in the tank, irradiance measures of the experimental tank were input into the von Kries’s simple color constancy model, (as in[39,40]). Irradiance measurements of the experimental tank were taken from the perspective of the male (*M*_*I*_—oriented slightly up and facing the female presentation container) and from the female perspective (*F*_*I*_—oriented toward the male nest, with the angle of the female throat). Two measures were taken from each point and averaged. Using equation 1 from Dalton *et al*. [40], the quantum catch (*Q*) of each eye receptor was calculated as: *K*

$$Q_{i}\;=\;K_{i}\int \;R_{i}\left(\lambda \right)\;L_{i}\left(\lambda \right)\;S_{i}\left(\lambda \right)\;I_{i}\left(\lambda \right)\;d\lambda$$

Terms in equation 1 include the cone sensitivity (*R*), the lens transmittance (*L*—which was assumed equal to one), the surface reflectance of the fish throat (*S*), and the irradiance measure of the light in the tank (*I*). The von Kries factor (*K*) is derived from equation 2 in Dalton *et al*. [40].

$$K_{i}\;=\;1/\left(\int \;R_{i}\left(\lambda \right)L_{i}\left(\lambda \right)I_{i}\left(\lambda \right)d\lambda \right)$$

*I* values in equation 1 were from the *F*_*I*_ irradiance measures, whereas in the calculation of *K*, *I* values were taken from the average of *M*_*I*_ and *F*_*I*_ irradiances so as to incorporate a ‘whole tank’ light assessment. Summations for each cone class (i.e. long, medium, short, UV), or absolute*Q* values, were calculated using cone sensitivity values from Rush *et al*. [41]Absolute *Q* values for each cone class were converted to relative quantum catches (*RQ*) by dividing each class by the sum of all other classes (For example: *RQ*_medium_ = *M / (UV+S+M+L)*). Corrected reflectance measures were used to calculate Cartesian coordinates, *x*, *y*, and *z*, with which throat color was calculated based on the Euclidean distance from the achromatic center in a tetrahedral color space[12,42,43]. The length of the vector from the origin to a point in the tetrahedral space, a measure of chroma[39,44], describes how much a color differs from achromatic black/white [44]. Measures of chroma have been commonly used to assess fish coloration [12,26,43,45,46] and have proven informative in studies of stickleback social interactions [25,47,48]. Our physiological model of stickleback vision covers the entire spectrum, including UV, which was supplemented during mate choice trials (see: ‘Fish collections and nesting stimulation‘). This allowed us to approximate color perception specific to sticklebacks [12], a method confirmed by a number of recent studies [39,41–43]. Colors of the same hue (found by measuring the angles the color point makes with the origin in space[39,44]) but different chroma will be distributed along one vector, with varying distances from the achromatic origin [44]. Unlike chroma, hue has not been shown to be informative in studies of stickleback social interactions. Therefore, hue was not used in this study.

#### Spine color

Pelvic spine color scores were determined from standardized photographs using *Adobe Photoshop CS3* as described in Yong *et al*. [12]. To calculate maximum spine coloration, the left spine was divided into eight equal, predetermined sections and the RGB values were recorded at the most intense red spot within each section. Individual RGB values were taken for each section, and standardized to an 18% gray card present in all pictures to obtain new, standardized RGB (R_Stand_, G_Stand,_ B_Stand_) values. Red spine intensity (*I*_*Red*_) for each section was then calculated by dividing R_Stand_ by the sum of R_Stand_, G_Stand_, and B_Stand_[12,31].

#### Body size

All females were measured for standard length (SL) at the conclusion of each presentation trial (as throat and spine color measurements were recorded), males at the conclusion of each presentation series.

### Statistical Analyses

#### Sequential choice trials

Principle components analysis (PCA) on the correlation matrix of male response variables was used to obtain a single composite variable of male mating behavior using ‘zigzags’, ‘bites’, ‘latency’, and ‘time in proximity’ to female. These four behaviors were chosen to account for the two common male courtship actions, ‘zigzags’ and ‘bites’, while also allowing for a measure of interaction time, ‘time in proximity’ to the female, as well as how quickly the male responded (moved one body length in the direction of the test female), ‘latency’. Values for zigzags, bites, and latency were log (x+1) transformed to improve normality. Raw ‘time in proximity’ scores displayed a relatively normal distribution and therefore were not transformed. Results of PCA using these four behaviors showed that PC1 loads positively with zigzags, bites, and time in proximity and negatively with latency (PC1: 51.9% of the variation; loading matrix shown in supplementary: S1 Table). PC1 therefore serves well as a composite male mate behavior (with a normal distribution). PC2 of the transformed data loads positively with bites, latency, and time in proximity, and negatively with zigzags (PC2: 21.8% of the variation; S1 Table), and therefore is indicative of a differential male response. PC1 of the transformed data was used in all subsequent analyses below and supplemented using PC2 and the individual behaviors underlying these composite traits (bites and zigzags).

Male mate preferences were estimated using a multivariate random-coefficient mixed model[49–53] fit via restricted maximum likelihood (REML) using the MIXED procedure in SAS v. 9.2 (SAS Institute, Cary, NC). Variation in male mating behavior (PC1 from above) was modeled as a linear function of the fixed effects of three continuous female traits: throat color, maximum spine color, and standard length. Additional fixed effects included the intercept, the continuous effect of trial number within a particular male (i.e. 1–5, as each male was presented with five different females in sequence), and the categorical effect of year (2010 or 2011). The categorical effect of time of a trial (morning vs. afternoon) was never significant in any model and was therefore excluded from analyses presented here. Following Dingemanse *et al*. [52], the female traits were individually standardized to zero mean prior to analysis such that the fixed-effect intercept captures variation in the elevation of the linear preference function, representing overall male responsiveness to females during their mating trials (i.e. PC1 score). Variances of the female traits were not transformed, although all results are qualitatively unchanged if variances are also standardized to one prior to analysis (H.D.R., unpublished results). The vector of fixed-effect partial-regression coefficients (i.e. slopes) for the three female traits in this model represent the average (i.e. population-level) male mate preference for each trait and are equivalent to the directional sexual selection gradients on them.

Variation in mate preference among individual males was specified by also including the intercept and three female traits as random effect terms in the model, thereby treating their coefficients as random samples from a population of possible coefficients (in this case, the population from which the males were drawn). In particular, the random-effect intercept represents among-individual variation in male responsiveness (PC1 score), or more precisely, the departure of the intercept for each male from the population-average, fixed-effect intercept. The random-effect partial regression coefficients for the three female traits characterize variation among males in the slope of their preference for each female trait (i.e. the departure of the regression slope for individual males from the respective population-average, fixed-effect estimate).

Testing of the random-effects employed a sequence of likelihood ratio tests (LRTs) in which-2 times the difference in log likelihood of two nested models provided a chi-squared distributed test statistic with degrees of freedom equal to the difference in the number of estimated (co)variance parameters between the two models. We did not model the covariances between the random effect intercept and slopes because their inclusion did not improve the fit of the model (LRT: χ^2^ = 0.475, d.f. = 3, P = 0.924). First, to provide a single overall test for variation in male mate preferences, we compared a model that included the random effect intercept and the full (i.e. six parameter), unconstrained covariance matrix for the random effect slopes with one that lacked all of these random effects. (Significance of all fixed effects described above was evaluated using this full model that included the random effects of both intercept and slopes.) Given significance of this overall test (see Results), we then separately tested for among-individual variation in slopes and intercept. Because effect significance can be sensitive to the failure to statistically accommodate variation that is present in the data, our test of the random effect intercept (slopes) compared models that included the random effect slopes (intercept) respectively. Partitioning variation into components due to slope and intercept can also be sensitive to the scaling of the independent variables (e.g., see[54]), and we therefore also present results using the raw (i.e. non-standardized) female traits, although the biological interpretations of the intercept vs. slope are less clear in this case.

Following McGuigan *et al*. [51] and Delcourt *et al*. [53], to test for variation in slope we employed a factor-analytical modeling approach that allows direct estimation of the eigenfunctions of the covariance function of random effect slopes[49,55,56]. This approach is analogous to the estimation and testing of the genetic principal components (i.e., eigenvectors) of an additive genetic covariance matrix[55,56]. To test the leading (first) eigenfunction, a LRT was used to compare a model specifying one dimension to the covariance matrix at this level (using the type = fa0(1)) option in the SAS Mixed procedure) to a model lacking these random effect slopes, with the random effect intercept being present in both of these models. To test the random-effect intercept, we compared models that included and excluded this term, with both models including the unconstrained random effect slopes.

The analyses above focus on linear mate preferences only. While more complex (e.g., second-order) functions can also be fit via random regression, we refrain from doing so here because, with 63 males, such a model would likely be over-parameterized. In addition, an exploratory analysis provides little evidence of nonlinear sexual selection (i.e. male mate preferences) at the population-level. In particular, treating every trial (very liberally) as independent, in a fixed-effect only model (fit via maximum likelihood) the addition of all the second-order interactions among the three female traits (i.e. both quadratic and correlational selection) did not significantly improve the fit of the model compared to one lacking these terms (LRT: χ^2^ = 3.4, d.f. = 6, P = 0.757), suggesting the absence of nonlinear mate preferences at the population level.

#### Simultaneous choice trials

PCA was again used on the correlation matrix of log (x+1) transformed values of zigzags, bites, and latency, extracting the primary axis of variation in male behavior. Time in proximity to the female could not be scored due to the perspective from which the simultaneous choice trials were recorded. PC1 again loaded positively with zigzags and bites and negatively with latency (PC1: 67.2% of the variation; S2 Table). PC2 loaded positively with zigzags and latency and negatively with bites (PC2: 20.2% of the variation; S2 Table).

Within each trial, females of a presentation pair were assigned to either a dull or red color category according to their individual throat color scores (higher color score assigned to the red category, lower score to the dull category). Male preference was assessed as the difference in male response directed toward each female as a function of the differences in each female trait (throat color, spine color, SL). For each presentation pair, male response difference (PC1) was calculated by subtracting the weaker male response from the stronger male response (always positive values). The differences in female traits were calculated by subtracting the trait score of the female who received the lower response from the female who received the stronger response (yielding both positive and negative values). PC2 differences in responses and trait scores were calculated in the same manner.

#### Conspecific male trials

In the same manner as the female sequential choice trials, PCA was used to obtain a composite male toward male behavior using log (x+1) transformed values of zigzags, bites, latency, and raw values of time in proximity. Results of PCA show that PC1 loads positively with zigzags, bites and time in proximity and negatively with latency (PC1: 57% of the variation, S3 Table). As with the female presentations, PC1 serves well as a summary indicator of male behavior. Because only one conspecific male was presented to each subject male, all of the trials were independent and standard first-order multiple regression was used to test the effect on the response of subject males of the intruder males throat color, spine color, and standard length. Subject males’ response (i.e. PC1 score) was also compared between trials using males and females, comparing the average male response in the five female presentations with male response to the ‘intruder’ male presentation.

## Results

### Sequential Choice Trials

**Fig 2:**
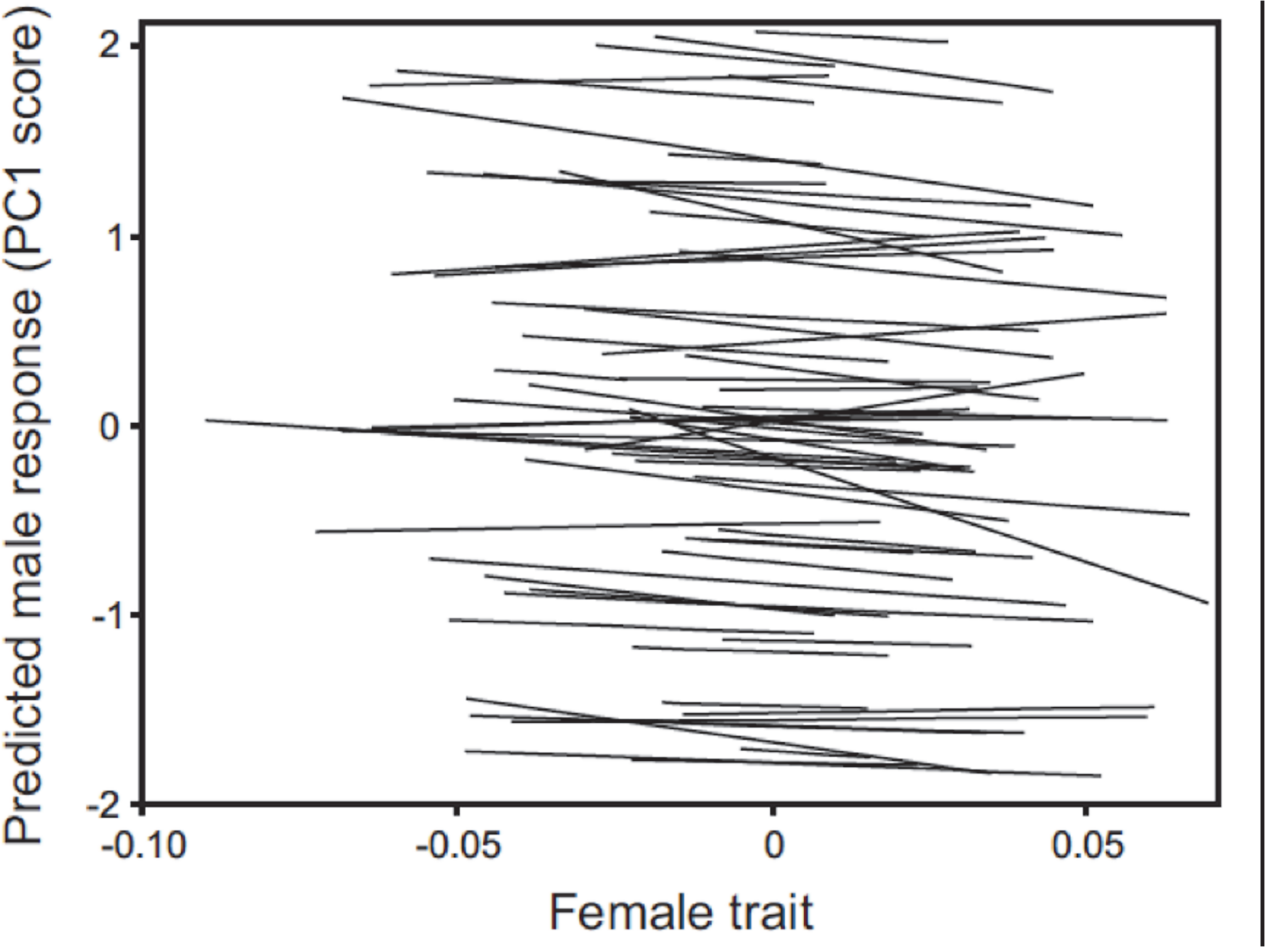
Predicted male mating response for female traits. Fitted mate preference function for each of the 63 males from the sequential choice mating trial assay, as estimated from a random coefficient mixed model (see Methods for details). Predicted values of the male mating response (PC1) are plotted against the composite trait values obtained by scoring females for the linear combination of the three traits (female throat color, spine color, standard length) described by the first eigenfunction of the random effect covariance function of male preference ([−0.210, −0.169, 0.005] for the three traits respectively).

There was no evidence of a population-level mate preference in males for female spine color or standard length (Table 1), suggesting the absence of any directional sexual selection on these traits arising from male mate choice. Male mating response (PC1) did increase with female throat color overall, suggesting possible sexual selection favoring females with redder throats, but the effect was not significant (P = 0.143; Table 1). At the individual level, significant among-male variation in mate preferences was detected overall (Fig. 2; LRT of random effect of intercept and slopes together: χ^2^ = 171.1, d.f. = 7, P < 0.0001). This appeared to arise from significant variation among males in their average responsiveness to females (LRT of the random effect intercept: χ^2^ = 169.3, d.f. = 1, P < 0.0001), but not from variation among males in the strength (i.e. slope) of their mate preferences as the leading eigenfunction of the covariance function of random effect slopes was non-significant (LRT: χ^2^ < 0.0001, d.f. = 3, P = 1.000). Given the absence of population-level preferences, this lack of significant variation among males in preference strength suggests the absence of individual-level preferences as well.

**Table 1:** Population-level male mate preferences.

Effect	Coefficient	F (d.f. = 1,62)	P
Female throat color	1.554	2.20	0.143
Female maximum spine color	−0.072	0.01	0.919
Female standard length	−0.012	1.51	0.224
Trial number	0.065	3.60	0.063
Year	NA	3.28	0.075

Model coefficients and significance (P) for the fixed effects on male responsiveness (PC1) of three female traits, trial number, and the categorical variable representing year of the trial (2010 vs. 2011). Fixed effects of the female traits would be indicative of population-level male mate preferences for them, and hence directional sexual selection on them.

Average male mating response (i.e. PC1 score) was higher in 2010 than 2011 and this difference approached significance (Table 1). There was also some evidence that males became more responsive to females in later trials (each male was tested with five females), suggesting perhaps that they were acclimating to the lab or test conditions, or that their desire to mate was increasing across trials. The effect, however, was marginally non-significant (Table 1). All of the above results, including those for population and individual-level male preferences, remain qualitatively unchanged if female traits are analyzed directly, without standardization to a mean of zero. The single exception is that the random effect intercept indicating variation in male responsiveness becomes marginally non-significant (χ^2^ = 2.74, d.f. = 1, P = 0.098).

Results from the analysis of PC2 followed the same general trend as those presented above. Significant among male variation in preference was detected (χ^2^ = 198.3, d.f. = 7, P < 0.0001) but this once again was attributed to significant variation among male intercepts (χ^2^ = 190.6, d.f. = 1, P < 0.0001) and not preference slopes (χ^2^ = 5.6, d.f. = 3, P = 0.1328). There is no evidence of population level preferences for throat color, spine color, or standard length (S4 Table). Unlike with PC1, PC2 responses did not differ between years. The order that females were presented, although non-significant, appears to have had an opposite effect as seen with PC1 with males decreasing slightly in response across multiple trails.

### Simultaneous Choice Trials

**Fig 3:**
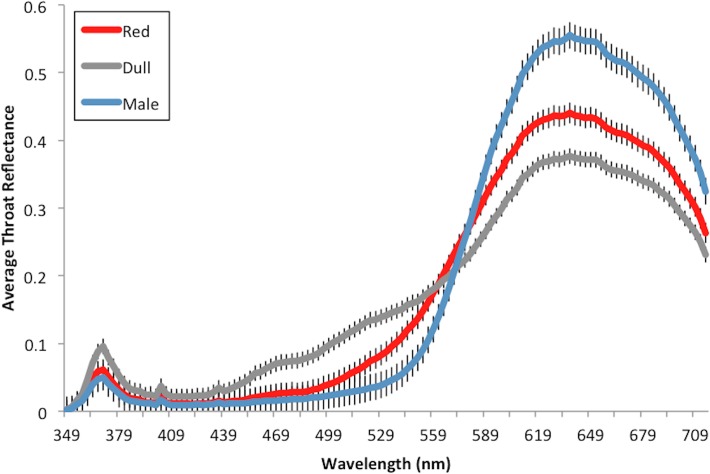
Average throat reflectance values of females used in simultaneous choice trials. A comparison of the average throat reflectance values of ‘dull’ versus ‘red’ categorized females used in simultaneous choice trials. Reflectance values were assessed in a tetrahedral color space model to obtain a single value for female throat color, a measure of chroma. Females were then assigned to a ‘red’ or ‘dull’ throat color category using this value, the highest value from each pair receiving the ‘red’ classification. Average subject male throat reflectance values included for comparison. Error bars represent ±1 S.E.

The pairs of females presented to a male differed significantly in throat color (t_31_ = 7.72, P < 0.001; Fig. 3) but not in standard length (t_31_ = 0.933, P = 0.3582). A one-way ANOVA shows that the differences in female traits (throat color, spine color, SL) had no effect on the difference in male response for either PC1 (F_3,28_ = 0.348, P = 0.791) or PC2 (F_3,28_ = 1.27, P = 0.303).

### Conspecific Male Trials

**Fig 4:**
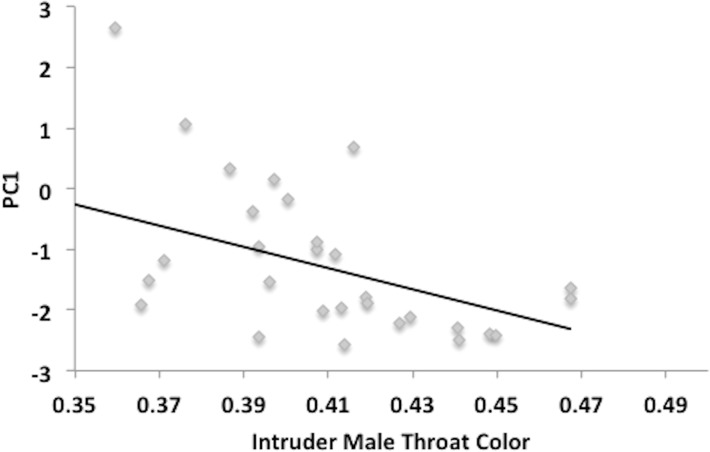
Intruder male throat color affects subject male behavioral response. Regression analysis of conspecific male trials shows that males display a significant, negative response to intruder male throat color (R^2^ = 0.188, df = 29, P = 0.016).

In a multiple regression analysis, male response (PC1) decreased significantly as intruder throat color increased (F_1,1_ = 6.868, P = 0.0145; Fig. 4). Male response (PC1) also decreased with increasing standard length of the intruder male, although the effect was marginally non-significant (F_1,1_ = 4.106, P = 0.053). Pelvic spine color (F_1,1_ = 1.729, P = 0.316) was non-significant. Variation in PC2 of the male response was unrelated to any of the intruder male traits (P > 0.3 in all cases).

**Fig 5:**
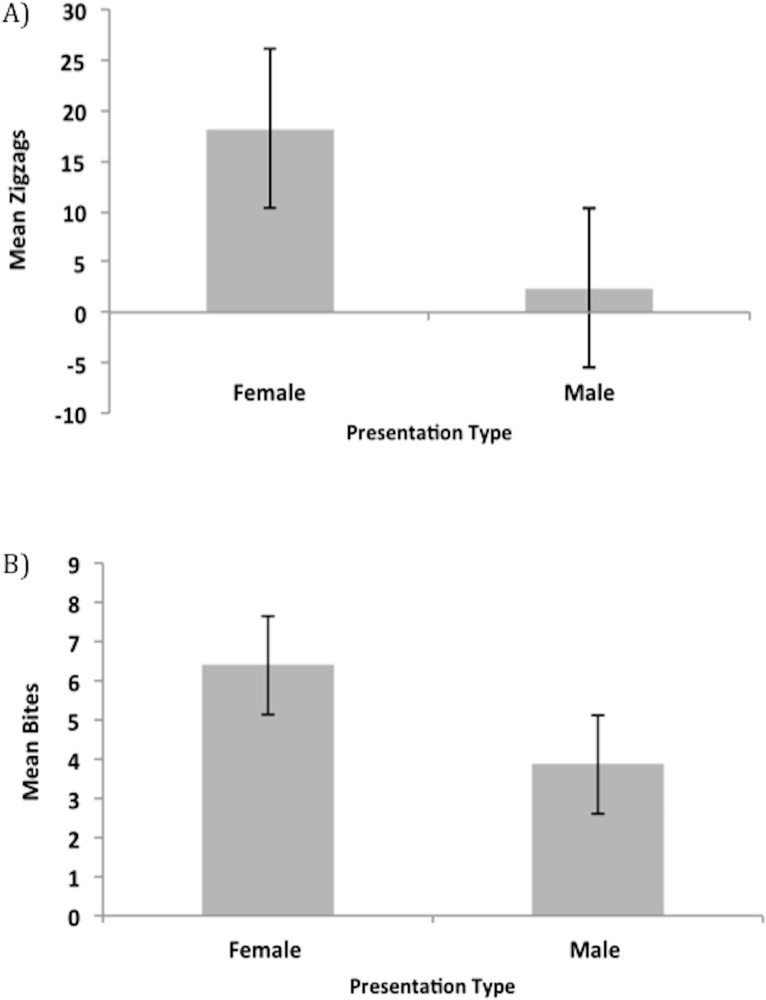
Male response differs between female and male presentations. Paired *t*-test revealed that in sequential choice trials, males (A) zigzagged significantly more toward females than males, but showed no difference in the number of (B) bites. Error bars represent ±1 S.E

Using only the 2011-year trials (male presentations were not performed in 2010), males were tested to see if they responded differently to females and males in the sequential choice presentations. One-way ANOVAs of PC1 and PC2 in 2011 female presentations show no differences in male response across trials (PC1: F_4, 155_ = 0.304, P = 0.875 & PC2: F_4, 155_ = 0.632, P = 0.64). Because males are clearly predicted to show differences in the zigzag courtship behavior toward males versus females, and to aid in interpretation of PC results, mean zigzags, and mean bites for comparison, were compared for the female and male presentations. Paired t-tests show that males displayed significantly more zigzags toward females (t_29_ = 4.933, P < 0.001, Fig. 5A) but showed no difference in the number of bites (t_29_ = 1.156, P = 0.2573, Fig. 5B).

## Discussion

Male threespine sticklebacks displayed no significant preference for female throat color in either simultaneous or sequential choice tests. In sequential trials, each male was presented with a series of females differing in throat color (and other traits). At the population level, average male response did show a trend of increasing in response to redder throats in females, but the effect was not significant, indicating the absence of sexual selection on these traits, and no variation in individual male preference strength was detected (although males did vary in the average responsiveness to females). In simultaneous trials, males were presented with two sized–matched, gravid females differing in throat color. Male response again did not differ for female throat color or pelvic spine color.

Using the sequential choice trials data, males were tested at the individual level for differences in their preferences for female traits. Results suggest that males vary in their responsiveness to females in general, but not in the strength of their preference for any of the female traits. When presented with a conspecific male in sequential choice tests during year two (in the same manner as females), males responded differently than in female presentations. Females received significantly more zigzags while bites between the two sexes did not differ. Also, male response decreased as the conspecific male throat coloration (and standard length, though non-significantly) increased. This shows that males could not only differentiate between males and females, but could also differentiate for traits of the fish being presented.

### Male Color Preference

The lack of male preference for female throat coloration is surprising given that females often display a strong preference for similar ornamentation in males (e.g. [23–25]) and female throat coloration must be conspicuous during the female “head up” behavior, adopted by receptive females. In other species where females possess ornamentation, male preference for brighter or more ornamented females is common, though far from ubiquitous [3]. In two-spotted gobies, males display a preference for female coloration [15], with female belly coloration being related to carotenoid levels in developing eggs. Similarly, male sockeye salmon preferentially select and spawn with females of redder hue, the predominant color in females [57]. The metric used to assess throat color in this study, chroma, has been used in a number of instances [12,26,43,45,46]and has proven informative in classic studies of stickleback social interactions[25,47,48]. Therefore, we feel our measurement of color is reliable and the lack of preference is attributable to another factor. Female sticklebacks used in this study displayed a significant correlation between body size (standard length) and red throat coloration [12]. Thus the lack of preference for throat color is also surprising (as well as the lack of a direct size preference) given the numerous examples of male fish preferring larger body size in females (discussed in more detail below).

One possible explanation for the absence of a male preference for female throat color in this study involves the operational sex ratio. The paternal care system of threespine sticklebacks’ means that males are investing heavily in each reproductive bout. Repeated periods of nest building, courtship, and egg guarding (and starvation due to not being able to forage) throughout a single breeding season can dramatically reduce the number of available males in the late breeding season. Therefore, if only in the late breeding period, the OSR could shift from male biased to female biased. A female biased OSR could encourage female-female competition for mates and males may stand to benefit by having some means to assess female quality. Observations by Kynard[34] and Mori[35] in stream and lake sticklebacks have shown females outnumbering males in the late breeding season, even to the point of multiple females attempting to court a single male. Should female throat color be an indicator of female quality, male preference for coloration may emerge in the latter parts of the breeding season, when males can afford to be choosy. Males used in this study were collected early in the breeding season, March 2010 and April 2011, so as to limit the effect of previous mating experiences and ensure reproductive readiness in trials. Early collection may have made the detection of male preference for female throat color difficult since the OSR may have not yet shifted. However, a large number of trials completed in this study did take place in the late breeding season (July-August 2010 & 2011). Additionally, males were tested in isolation, without competition from rival males. Females were presented sequentially, varying in an array of traits, and simultaneously, in size-matched pairs differing in throat color. Therefore, potential OSR effects that could be encountered in the late breeding season were implemented in this study (i.e. females out numbering males and low male-male competition). Even with these factors, no preference was observed. Mate choice tests of this population in a more natural setting may prove beneficial to exploring male preference in the late breeding season, as well as direct observations.

An alternative explanation to the results of this study may involve the fact that fish were tested in clear water conditions, with ample illumination and a full spectrum of light, including UV. This was done so as to minimize the effects of environmental conditions on male perception of female throat color (and to likewise maximize the transmission of the female signal). Additionally, females were presented in a stable, head-up position, with males often approaching from in front of and below the female. However, water from the upper-stream Little Campbell is tea-stained in color (personal observation—DSW) and in natural mating encounters, males are likely to view females (and their throat color) from an array of perspectives and distances. Both of these factors, watercolor and the male/female spatial relationship, could have an effect on color perception (for example: viewing females from below with the sky as background vs. viewing the female on an even plane). Once again, testing under more natural conditions (i.e. tea-stained water and full interaction) may prove beneficial in exploring this topic further.

Since males display no preference for throat coloration in females, female throat color in this population may not be sexually selected. Instead, female throat color may arise through a genetic correlation with the phenotype under sexual selection in males[13]. Having derived from a marine population lacking female throat color[27] the upper-stream Little Campbell may differ significantly from lower-stream and marine environments. In particular, factors such as lower predation levels, differential food supplies, and stained water may work to relax selection against female coloration. Should there be lower costs associated with the trait, females could ‘afford’ to be colorful. Weakened selection pressure for dichromatism could therefore explain why female throat color is so common in this population despite being largely absent elsewhere. Thus selection might be important, but more in terms of the cost of the trait than in its benefits.

### Male Size Preference

Males displayed no population level preference for female body size in sequential presentations (size preference in simultaneous presentations was not tested since females were presented in size-matched pairs). The lack of male preference for female body size is unexpected given the numerous examples of male preference for larger females across fish species. Studies with sticklebacks[58–60], mosquito fish[61], guppies[62], and blennies[63] have all shown evidence of male fish preferring larger females. This preference is thought to stem from the fact that larger female body size is typically correlated with increased fecundity[64], a trend present in experimental females used in this study (Wright, unpublished). However, work by Dosen and Montgomerie[62] did show that, although non-significant, male guppies spent slightly more time with smaller females when the size difference between females was less than 4mm. A similar trend was observed in convict cichlids, with males preferring the smaller female more often when SL differences were less than 5mm[65]. No such trends are apparent with males in this study.

In the absence of preference for female size, it is reasonable to question if the experimental design allows for males to properly assess relevant traits of presented fish. The conspecific male trials completed during the second year indicate that the design does permit such assessment. Males presented with a conspecific, same sex intruder responded differently relative to when females were presented in the same manner. Significantly more zigzags (indicating courtship) were directed towards females while the number of bites (which occur in both courtship and aggression) did not differ. Additionally, as intruder male standard length and throat color increased, male response (PC1) decreased. These results suggest that males were able to not only distinguish between males and females in the experimental set-up, but could also differentiate body size and color. It is also possible that power in this study was inadequate to detect preferences, but the significant results with regard to stimulus sex and male traits argue against this interpretation.

### Male Spine Color Preference

Males displayed no population level preference for female pelvic spine coloration, in contrast to Nordeide[30], who found that males directed more courtship to females of drab pelvic spine color when presented in simultaneous pairs. The lack of preference for spine color in this experiment may stem from the fact that females were lightly anesthetized during presentations, meaning that pelvic spines were not erect, perhaps making male assessment of spine color difficult (although spine color is evident when fish are viewed from the ventral side: DSW, personal observation). The correlation between female spine color and female throat color (r = 0.35, S1 Fig., [12]) could also explain the lack of spine color preference—conceivably, preference for one and aversion for the other could cancel out each effect to some degree.

Unlike throat coloration, the presence of pelvic spine color in females may not be as easily explained as a correlated response to sexual selection in males. Being that very few studies have addressed this trait specifically [30,31,66], one can only speculate about the evolutionary origin of this ornament. Nordeide[30] found that males preferred drab spine coloration in females, suggesting a non-adaptive correlation. Additionally, Nordeide[31] found that female spine color displayed a negative association with carotenoid levels in the gonads, a relationship that is currently being explored in females of our study population.

### Conclusion

Working from the knowledge that red throat coloration in male threespine sticklebacks is often a sexually selected trait; the same ornament in females might be predicted to be under similar selective pressure. While natural observations and field studies can certainly help to further explore this hypothesis, the results of this study suggest that red throats in females are not under strong male mate preference. However, the existence of female coloration still poses an intriguing problem, especially since the trait has also been observed in females from other populations. Preference tests in other populations could help to further our understanding of the role sexual selection plays in this traits’ existence and assess the generality of our findings.

## Supporting Information

S1 Fig Regression of experimental females' throat color and pelvic spine color (R^2^ = 0.125, P < 0.001) (TIFF)

S1 Table Loading matrix for principal component analysis of sequential choice trials (DOCX)

S2 Table Loading matrix for principal component analysis of simultaneous choice trials (DOCX)

S3 Table Loading matric for principal component analysis of conspecific male trials (DOCX)

S4 Table Model coefficients and significance (P) for the fixed effects on male responsiveness (PC2) of three female traits, trial number, and the categorical variable representing year of the trial (2010 vs. 2011). (DOCX)
