# Supplementary figures and images for: Conspicuous Female Ornamentation and Tests of Male Mate Preference in Threespine Sticklebacks (Gasterosteus aculeatus)
Source: PLoS One. 2015 Mar 25;10(3):e0120723. doi: 10.1371/journal.pone.0120723 (PMC4373685; doi:10.1371/journal.pone.0120723)

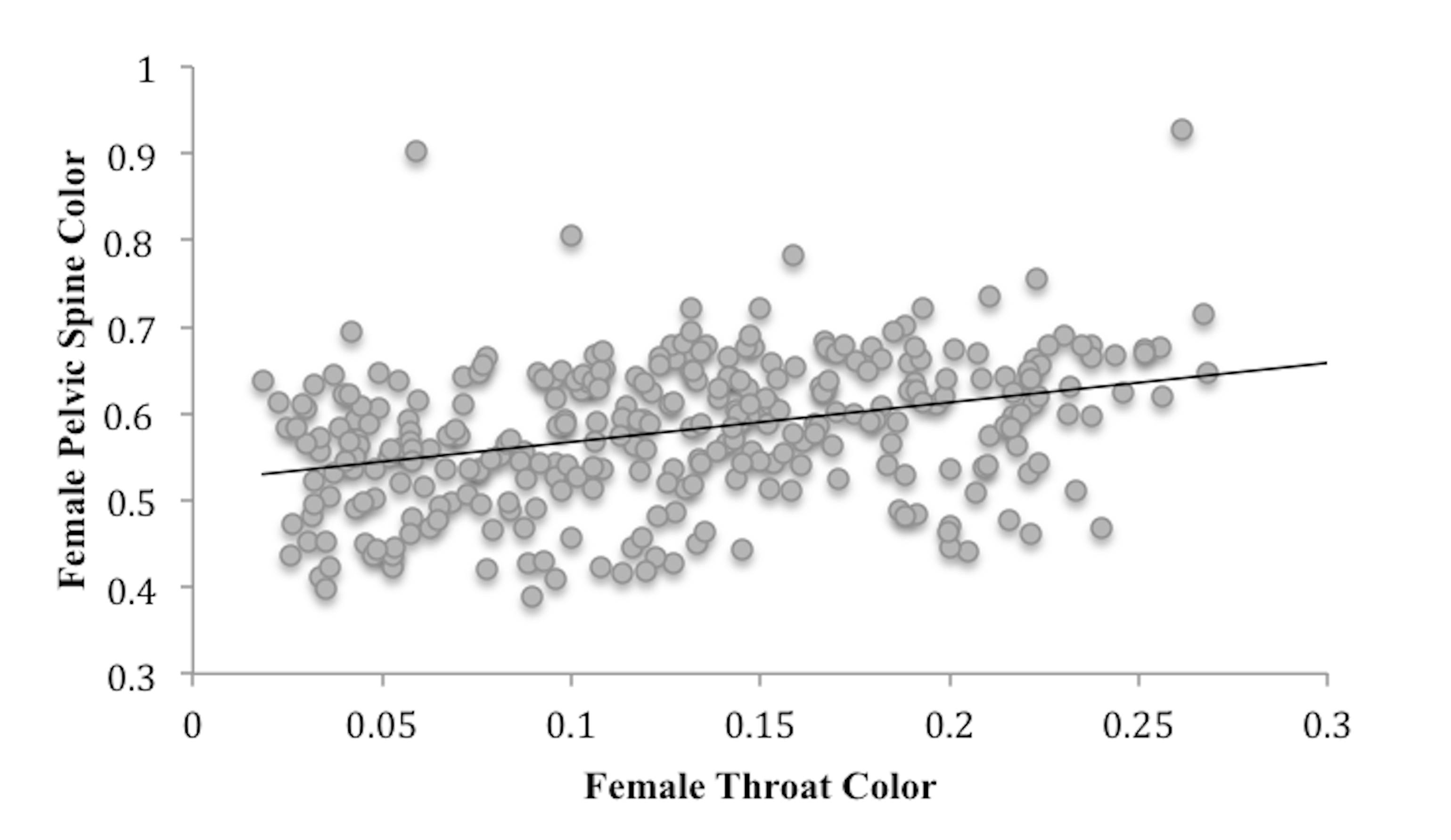

Supplement: S1 Fig — (TIFF) [file pone.0120723.s001.tiff]
