# Supplementary material for: Conspicuous Female Ornamentation and Tests of Male Mate Preference in Threespine Sticklebacks (Gasterosteus aculeatus)
Source: PLoS One. 2015 Mar 25;10(3):e0120723. doi: 10.1371/journal.pone.0120723 (PMC4373685; doi:10.1371/journal.pone.0120723)
Supplement: S1 Table — (DOCX) [file pone.0120723.s002.docx]

| Male response variables | PC1 | PC2 |
| --- | --- | --- |
| Log (ZZ+1) | 0.618 | -0.539 |
| Log (Bites+1) | 0.774 | 0.498 |
| Log (Latency+1) | -0.622 | 0.491 |
| In Proximity | 0.842 | 0.303 |
|  |  |  |
|  | | |
