# Supplementary material for: Conspicuous Female Ornamentation and Tests of Male Mate Preference in Threespine Sticklebacks (Gasterosteus aculeatus)
Source: PLoS One. 2015 Mar 25;10(3):e0120723. doi: 10.1371/journal.pone.0120723 (PMC4373685; doi:10.1371/journal.pone.0120723)
Supplement: S2 Table — (DOCX) [file pone.0120723.s003.docx]

|  |  |  |
| --- | --- | --- |
|  |  |  |
| Male response variables | PC1 | PC2 |
| Log (ZZ+1) | 0.750 | 0.661 |
| Log (Bites+1) | 0.851 | -0.304 |
| Log (Latency+1) | -0.855 | 0.278 |
|  |  |  |
|  | | |
|  |  |  |
