# Supplementary material for: Conspicuous Female Ornamentation and Tests of Male Mate Preference in Threespine Sticklebacks (Gasterosteus aculeatus)
Source: PLoS One. 2015 Mar 25;10(3):e0120723. doi: 10.1371/journal.pone.0120723 (PMC4373685; doi:10.1371/journal.pone.0120723)
Supplement: S3 Table — (DOCX) [file pone.0120723.s004.docx]

| \| Male response variable \| PC1 \| PC2 \| \| --- \| --- \| --- \| \| Log (ZZ+1) \| 0.693 \| -0.183 \| \| Log (Bites +1) \| 0.85 \| 0.401 \| \| Log (Latency+1) \| -0.488 \| 0.818 \| \| In Proximity \| 0.916 \| 0.202 \| \|  \|  \|  \| \|  \| \| \| \| |
| --- | --- | --- | --- | --- | --- | --- | --- | --- | --- | --- | --- | --- | --- | --- | --- | --- | --- | --- | --- | --- | --- |
