# Supplementary material for: Conspicuous Female Ornamentation and Tests of Male Mate Preference in Threespine Sticklebacks (Gasterosteus aculeatus)
Source: PLoS One. 2015 Mar 25;10(3):e0120723. doi: 10.1371/journal.pone.0120723 (PMC4373685; doi:10.1371/journal.pone.0120723)
Supplement: S4 Table — (DOCX) [file pone.0120723.s005.docx]

| Effect | Coefficient | F Value (d.f.=1,62) | P |
| --- | --- | --- | --- |
| Female throat color | 0.065 | 0.01 | 0.934 |
| Female maximum spine color | -0.643 | 1.77 | 0.189 |
| Female standard length | -0.005 | 0.76 | 0.386 |
| Trial number | -0.037 | 3.20 | 0.079 |
| Year | NA | 0.76 | 0.386 |
|  |  |  |  |
|  | | | |
|  |  |  |  |
|  |  |  |  |
